# VOC Emission Reduction and Rheological Optimization of Recycled Asphalt with USP Warm Mix Additive

**DOI:** 10.3390/polym18162022

**Published:** 2026-08-20

**Authors:** Zhaoyang Wang, Bowen Guan, Xuetao Wang, Anhua Xu, Xin Zheng, Yue Zhang

**Affiliations:** 1 Qinghai Provincial Key Laboratory of Tibet Plateau Highway Construction and Maintenance Technology, Qinghai Polytechnic University, Xining 810003, China; 2024231043@chd.edu.cn; 2 School of Materials Science and Engineering, Chang’an University, Xi’an 710061, China; 2023231093@chd.edu.cn; 3 Qinghai Provincial Transportation Holding Group Co., Ltd., Xining 810002, China; 13618328184@163.com; 4 School of Intelligent Science and Engineering, Xi’an Peihua University, Xi’an 710125, China; zhengxin@peihua.edu.cn

**Keywords:** USP warm mix additive, waste soybean oil recycled asphalt, VOCs emission, rheological properties, inhibition mechanism, multi-performance balance

## Abstract

To mitigate high-temperature VOC emissions and reduce construction temperatures in recycled asphalt, a USP warm mix additive was introduced into waste soybean oil (WSO) recycled asphalt. This study systematically investigates the effects of USP content (1%, 3%, 5%, and 7%) and construction temperature (140 °C and 160 °C) on VOCs emission characteristics, inhibition mechanisms, and rheological performance. The results show that USP reduces total VOC emissions, with the key inhibition effect achieved at 5% USP. At 160 °C, the VOCs inhibition rate reached 39.5% at 5% USP, while at 140 °C it increased to 80.8%; the findings suggest that lower temperatures enhance the inhibitory effect. The results show USP cuts emissions via physical mechanisms. However, the significant FTIR and DSC analyses suggest that VOC reduction appears to be primarily governed by physical mechanisms, including phase-change cooling, physical encapsulation, and migration retardation, without altering the chemical structure of asphalt. Moreover, the important rheological results indicate that although USP slightly decreases the high-temperature complex modulus and rutting factor, the evidence demonstrates that it improves the percent recovery (R_0.1_ from 53% to 66%; R_3.2_ from 21% to 40%) and low-temperature crack resistance through decreased stiffness S and increased m-value. In light of these significant findings, the study demonstrates that USP exhibits a multi-performance balance, appearing to reduce VOC emissions and improve workability while moderately weakening high-temperature deformation resistance. Notwithstanding the reduced high-temperature resistance, the key evidence could demonstrate that USP enhances elastic recovery and low-temperature performance in the results. The optimal USP content appears to be 5%, providing the critical compromise between emission reduction and pavement performance for WSO recycled asphalt.

## 1. Introduction

As a major component of global transportation infrastructure, asphalt pavement plays an irreplaceable role in modern highway construction. However, during high-temperature construction and service, asphalt materials release large quantities of volatile organic compounds (VOCs) [1], which not only pose a serious threat to the health of construction workers but also cause significant pollution to the surrounding atmospheric environment [2]. In recent years, with the growing global awareness of environmental protection and the increasingly stringent environmental regulations worldwide, VOC emissions during asphalt pavement construction have become a key environmental issue that urgently needs to be addressed in the field of road engineering. Studies have shown that VOCs released from asphalt at high temperatures mainly include polycyclic aromatic hydrocarbons (PAHs), alkanes, benzenes, and other hazardous substances [3]. These compounds are carcinogenic and mutagenic, and long-term exposure can lead to respiratory diseases and even cancer [4]. Therefore, how to effectively reduce VOC emissions during construction while ensuring the performance of asphalt pavement has become a hot and difficult topic in current asphalt materials research.

At the same time, the resource utilization of waste cooking oil has become an important component of circular economy and sustainable development strategies. As one of the largest consumers of edible oil in the world, China generates millions of tons of waste cooking oil annually. If this waste oil is not properly disposed of, it not only results in huge resource waste but also causes severe pollution to soil and water bodies. The recycling and reuse of waste cooking oil in the field of road engineering, particularly as a rejuvenator for recycled asphalt [5], has been proven to be a feasible approach with both economic and environmental benefits. Waste soybean oil is rich in unsaturated fatty acid glycerides, which endow it with good softening and rejuvenating abilities for asphalt. It can effectively restore the rheological properties of aged asphalt and improve the ductility and low-temperature crack resistance of recycled asphalt [6]. Su et al. [7] used chitosan/sodium alginate (CS/SA) as microcapsule shells to encapsulate waste soybean oil, achieving a self-healing efficiency of over 60% with 4 wt% microcapsules, which is three times that of neat asphalt. Zheng et al. [8] rejuvenated aged asphalt with modified waste cooking oil (MWCO). Through microscopic characterization combined with statistical analysis, they demonstrated that MWCO not only reversed the phase separation caused by the volatilization of maltene components in traditional aged asphalt but also enhanced the compatibility among components via polar ester groups. Ye et al. [9] reduced the fatty acid content of waste cooking oil (WCO) in advance through catalytic esterification technology and experimentally proved that recycled asphalt containing 6% WCO exhibited superior high-temperature rheological properties compared to virgin asphalt. However, waste soybean oil recycled asphalt also faces the problem of high VOC emissions during high-temperature construction. Because waste soybean oil itself contains a large amount of volatile fatty acid components, it is prone to vaporization under high-temperature construction conditions [10], leading to severe VOC emissions from waste soybean oil recycled asphalt mixtures during construction, thus further exacerbating the contradiction between green recycling and environmental protection.

To address the above issues, warm mix asphalt technology provides a promising solution. By incorporating warm mix additives [11], warm mix technology enables asphalt mixtures to be mixed and compacted at lower temperatures, thereby reducing the generation and emission of VOCs at the source. Currently, common warm mix additives mainly include surfactants, organic waxes, foaming agents [12], and chemical additives. Among them, the USP warm mix additive, as a novel multifunctional warm mix material [13], has received widespread attention in recent years. The USP additive is mainly composed of inorganic minerals and special functional components, which can significantly reduce the construction viscosity of asphalt through physical mechanisms, achieving the goal of low-temperature construction. Compared with traditional organic warm mix additives, USP offers advantages such as good environmental friendliness, no introduction of additional organic pollutants, and strong compatibility with various asphalt systems. Shi et al. [14] studied the performance of USP warm mix rubber asphalt and found that the addition of the USP warm mix additive increased the penetration, ductility, and softening point of rubber asphalt while effectively improving its low-temperature performance. Liu et al. [15] incorporated the USP warm mix additive into 70# base asphalt and SBS-modified asphalt for comparison. Rheological test results showed that USP improved the deformation resistance and creep recovery capacity of the asphalt without causing chemical reactions; however, excessive USP addition led to changes in the spatial network structure of the asphalt, resulting in reduced high-temperature performance. Nevertheless, research on the application of USP additives in waste cooking oil recycled asphalt systems remains very limited, and the inhibitory effect of USP on VOC emissions from waste soybean oil recycled asphalt, as well as its mechanism of action, is still unclear.

In terms of asphalt rheological properties, controlling VOC emissions often comes at the cost of sacrificing some high-temperature performance [16]. The high-temperature rheological properties of asphalt [17] (e.g., complex modulus, rutting factor, etc.) are key indicators for evaluating the rutting resistance of pavements, while low-temperature performance [18] (e.g., creep stiffness, m-value, recovery rate, etc.) determines the cracking resistance of pavements. The rheological behavior of asphalt undergoes complex changes after the addition of warm mix additives and rejuvenators [19]: on one hand, the introduction of rejuvenators can improve the low-temperature performance of aged asphalt [20]; on the other hand, the temperature-reducing effect of warm mix additives may lead to a decrease in asphalt stiffness at high temperatures, thereby weakening its deformation resistance [21]. Therefore, achieving a performance trade-off relationship among reducing VOC emissions, improving low-temperature performance, and maintaining sufficient high-temperature rutting resistance is a core challenge in current research on recycled warm mix asphalt.

In summary, against the backdrop of the “carbon peak and carbon neutrality” goals and the development of green transportation, introducing the USP warm mix additive into the waste soybean oil recycled asphalt system and systematically investigating its inhibitory effect on VOC emissions, the underlying mechanism, and the comprehensive influence on the rheological properties of asphalt not only holds significant theoretical importance for promoting the high-value utilization of waste cooking oil and the green construction of asphalt pavements, but also offers considerable practical value for guiding the engineering application of waste soybean oil recycled warm mix asphalt. It is in this context that the present study was carried out. The aim is to reveal the effects of USP content and construction temperature on the VOC emissions and rheological properties of waste soybean oil recycled asphalt through systematic experiments and multi-dimensional analysis, and to determine the optimal USP dosage, thereby providing a scientific basis for the multi-performance balance design of waste soybean oil recycled warm mix asphalt.

## 2. Materials and Methods

### 2.1. Raw Materials

#### 2.1.1. Asphalt and WSO

The experimental asphalt used was Zhenhai Refinery Donghai Brand 70# A-grade highway petroleum asphalt. The asphalt was tested according to the JTGE20-2011 standard [22], and the properties are listed in Table 1.

The waste soybean oil used in this study was sourced from the domestic Chinese market and was heated in an oven at 100 °C for 2 h prior to use to remove moisture. The basic physical properties of the WSO are listed in Table 2.

#### 2.1.2. USP Warm Mix Additive

The USP warm mix additive used in this study was purchased from China Oil Luchixing Material Co., Ltd. (Zhengzhou, China).Its main technical performance indicators are listed in Table 3.

### 2.2. Test Methods

#### 2.2.1. Preparation of Aged Asphalt

Based on JTGE20-2011, the 70# base asphalt was placed in a rotating thin film oven at 163 °C for aging for 85 min. The sample after rotating thin film oven aging was poured into a stainless steel pan and placed in a PAV pressure aging vessel. The aging temperature was 100 °C, and the air pressure was (2.1 ± 0.1) MPa. The test duration was 20 h. After the specified time, the pressure inside the aging vessel was reduced to atmospheric pressure within 8 to 15 min to obtain the aged asphalt. Some performance indicators of the aged asphalt are listed in Table 4.

#### 2.2.2. Preparation of Recycled Asphalt

The aged asphalt and waste soybean oil were heated at 135 °C and 100 °C for 2 h, respectively. The aged asphalt was then mixed with waste soybean oil at a content of 8%. After that, the mixture of waste soybean oil and aged asphalt was preheated to 135 °C. High-speed shearing was performed at 140 °C for 60 min with a shear rate of 3000 r/min. After cooling, the recycled asphalt was obtained [23]. After cooling, the recycled asphalt was named WSO-recycled asphalt and designated as 8% WSO.

#### 2.2.3. Preparation of Warm Mix Recycled Asphalt

Using the USP warm mix additive, different dosages of USP (1%, 3%, 5%, 7%) were placed in an oven at 120 °C for 20 min to melt into a fluid state. Meanwhile, the waste soybean oil recycled asphalt was heated to 150 °C. To ensure temperature stability during heating, the oil bath temperature was adjusted to 180 °C. The molten USP warm mix additive was gradually added to the waste soybean oil recycled asphalt while continuously stirring with a glass rod. Finally, the mixture was sheared at a high speed of 4000 r/min for 120 min, and then placed in an oven at 150 °C for 30 min to obtain the USP warm mix modified recycled asphalt.

FTIR analysis confirmed that the carbonyl index C=O/(C–H) changed negligibly from 0.042 to 0.044 before and after shearing, indicating that oxidative aging was effectively controlled. All samples were subjected to identical shearing conditions to ensure valid relative comparisons.

#### 2.2.4. Asphalt VOC Collection Experiment

**Figure 1:**
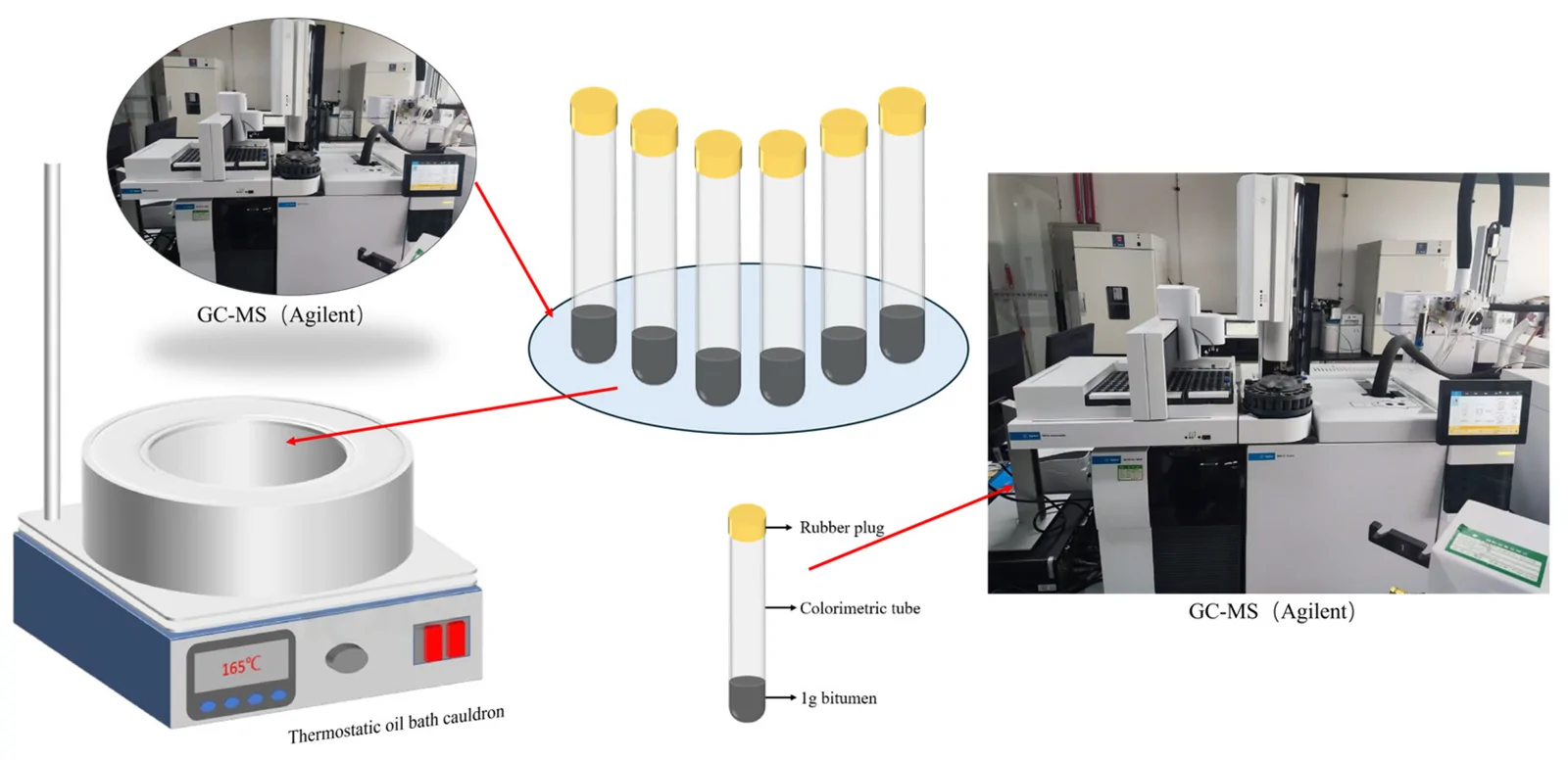
Asphalt VOC heating experiment.

**Figure 2:**
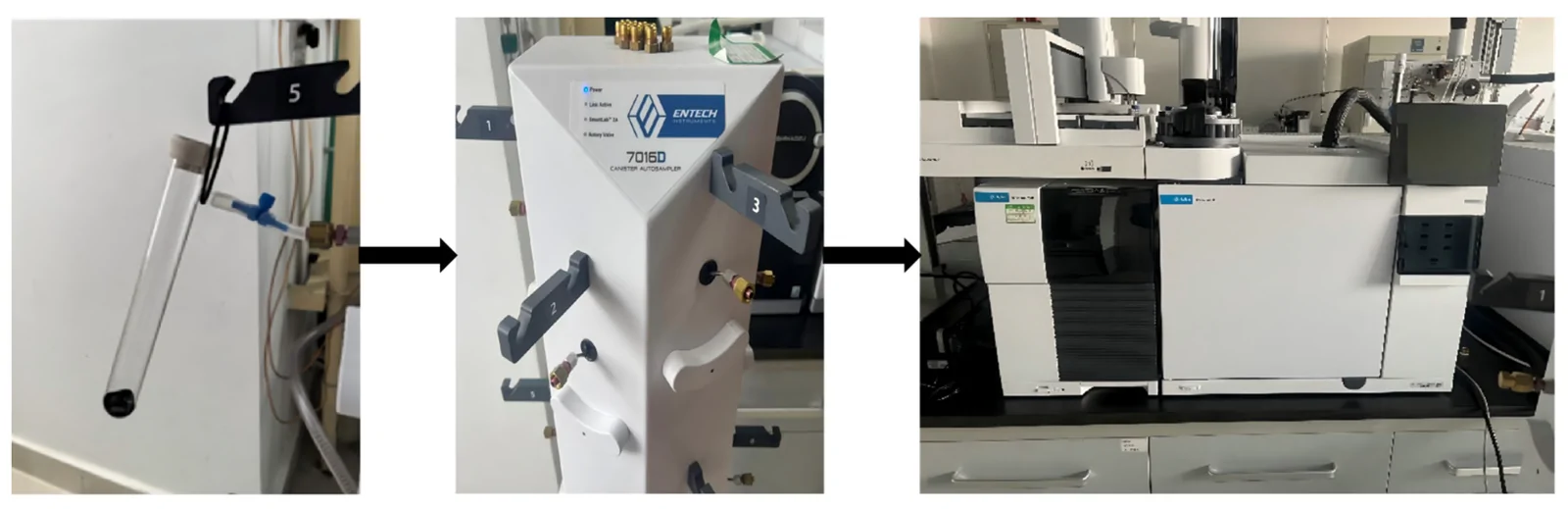
Gas chromatography-mass spectrometer (GC-MS).

Asphalt mixtures emit a large amount of VOCs during use. This study designed related experiments to simulate this process. Referring to other similar studies [24,25,26], the heating temperature for the simulation experiment was set at 165 °C, with insulation times chosen as 30 min [27]. To ensure temperature stability during the insulation process, an oil bath heater was selected. The setup of the simulation experiment is shown in Figure 1 and Figure 2.

One gram of asphalt sample was placed in 100 mL volumetric colorimetric tubes, with the asphalt sample positioned at the bottom of the tube to ensure uniform heating. The inner walls and mouth of the tube should be free of asphalt samples to prevent instrument contamination and reduce errors. The colorimetric tubes were placed in the oil bath and heated to 165 °C at a rate of 4 °C/min. After reaching 165 °C, five colorimetric tubes were removed after 30 min of insulation. The VOC components and their concentrations in six tubes were determined using gas chromatography-mass spectrometry (GC-MS).

#### 2.2.5. Asphalt VOC Testing Method

VOCs were qualitatively and quantitatively determined using a gas chromatography-mass spectrometer (Agilent 8890-5977B, Agilent Technologies, Inc., Santa Clara, CA, USA). The ion source temperature was maintained at 230 °C, and the MS detector was operated in full scan electron ionization (EI) mode, collecting data within the *m*/*z* 35–200 range.

During the result screening process, VOCs with concentrations greater than 0 and spectral library matching degrees greater than 80 were selected as criteria, ultimately identifying 61 VOCs as quantitative research objects. Based on molecular structural characteristics and toxicity differences, these were classified into five categories: alkanes, alkenes, hydrocarbon derivatives, aromatic hydrocarbons, and sulfur-containing compounds.

To evaluate the effect of asphalt VOC emissions, the VOC inhibition rate was introduced as a characterization indicator, with the calculation formula as follows:

VOC inhibition rate (%) = (C_0_ − C_1_)/C_0_ × 100% (1)

where C_0_ is the VOC concentration of the original asphalt, and C_1_ is the VOC concentration of the modified asphalt.

The baseline VOC concentration C_0_ used in the inhibition rate calculation was obtained by averaging the total VOC concentrations from three replicate experiments of the 8% WSO sample without USP at each respective temperature.

All experiments were conducted in triplicate (*n* = 3), and results are reported as mean ± standard deviation (SD). One-way ANOVA with Tukey’s post hoc test (*p* < 0.05) was used for statistical comparisons. Error bars in all figures represent the standard deviation calculated from three independent replicate measurements.

#### 2.2.6. Fourier Transform Infrared (FT-IR) Analysis

The tests were conducted in the scanning range of 400 cm^−1^ to 4000 cm^−1^ with a resolution of 4 cm^−1^. Asphalt samples were dissolved in a 5% CS_2_ solution. Subsequently, the sample solution was dropped onto a KBr plate for FT-IR analysis.

#### 2.2.7. Differential Scanning Calorimetry (DSC)

DSC was used to examine the temperature stability of the USP warm mix additive. Moreover, the significant findings could indicate that approximately 10 mg of the sample was weighed and placed into an aluminum crucible. In light of the experimental evidence, the crucible demonstrates that heating from 80 °C to 200 °C at a heating rate of 10 °C/min, with air flowing at 50 mL/min as the protective gas, could demonstrate reliable thermal analysis. Furthermore, the results might indicate that these conditions appear critical for accurate measurement. DSC shows stability across the temperature range.

#### 2.2.8. Dynamic Shear Rheometer (DSR) Test

The rheological properties of the recycled asphalt demonstrate that a dynamic shear rheometer (DSR) could be the most relevant analytical approach. However, the significant findings could indicate that the complex modulus (G*) and phase angle (δ) of the modified asphalt appear critical over a temperature range of 40 °C to 80 °C with a precise heating rate of 2 °C/min. Given that the experimental evidence demonstrates that the rotor diameter of 25.0 mm and a sample thickness of 1.0 mm appear significant, the results might indicate that these parameters could support reliable measurement. Therefore, the key data demonstrate that the test conditions appear carefully controlled at a frequency of 10 rad/s and a strain of 0.5%. DSR results show G* and δ characterize asphalt mastic rheology.

#### 2.2.9. Multiple Stress Creep Recovery (MSCR) Test

The study could indicate that multiple stress creep recovery (MSCR) tests, referring to standard AASHTO TP70, may demonstrate that the evidence appears to support reliable specimen analysis. Additionally, the significant results might suggest that the core parameters could indicate that at the temperature corresponding to the high-temperature performance grade (PG) of the asphalt, typically 64 °C, the findings appear relevant. Notwithstanding the variability in specimen response, the key evidence demonstrates that repeated creep loads at two stress levels of 0.1 kPa and 3.2 kPa could demonstrate that sequential application appears important. Nevertheless, the data might indicate that these conditions support the conclusion that the results appear consistent with established performance criteria. MSCR shows that creep loads affect asphalt specimens at stress levels. Each creep loading phase lasted 1 s, immediately followed by a 9 s unloading recovery phase, forming a 10 s cycle. At least 20 effective cycles were performed at each stress level (typically, the first 10 cycles were used for specimen conditioning, and the subsequent 10 cycles were used for data collection) to calculate the non-recoverable creep compliance (Jnr) and the average percent recovery (R) at each stress level. The test was conducted in strain-controlled mode, with a data acquisition frequency typically not less than 10 Hz, to obtain accurate creep and recovery curves.

#### 2.2.10. Bending Beam Rheometer (BBR) Test

The BBR was used to study the low-temperature cracking performance of asphalt. When the load was applied to the asphalt binder beam for 60 s, the creep stiffness (S) and m-value of the asphalt were obtained, which describe the beam’s resistance to loading and the low-temperature creep rate, respectively. The test temperatures were −12 °C, −18 °C, and −24 °C.

## 3. Results and Discussion

### 3.1. Effect of USP Warm Mix Additive on VOC Emission from Recycled Asphalt

#### 3.1.1. VOC Release Pattern at the Same Temperature

**Figure 3:**
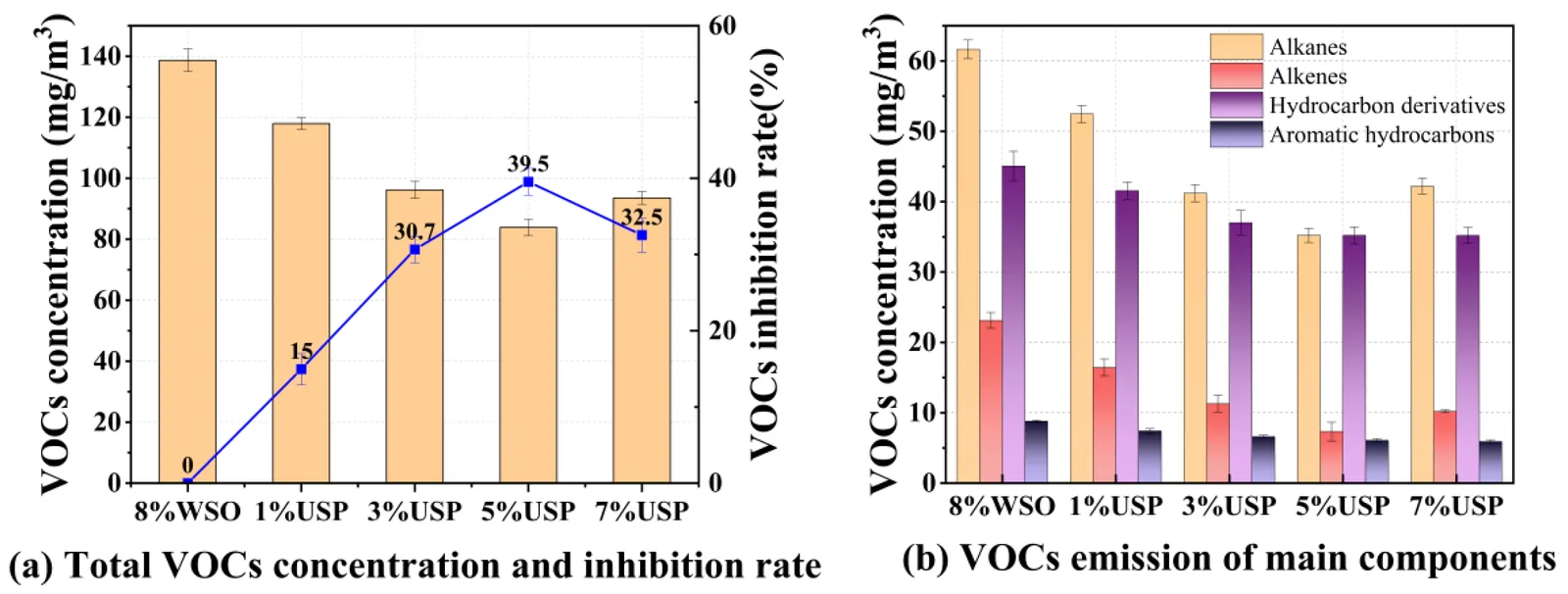
Total VOC emission pattern of USPRA at 160 °C.

At 160 °C, after incorporating USP into the 8% WSO recycled asphalt, the total VOC emission of the system exhibited a trend of first decreasing and then slightly increasing. As shown in Figure 3a, without USP, the 8% WSO sample had the highest total VOC concentration, approximately 139 mg/m^3^. As the USP content increased from 1% to 5%, the total VOC concentration continuously decreased to approximately 118, 96, and 84 mg/m^3^, corresponding to VOC inhibition rates of 15.0%, 30.7%, and 39.5%, respectively. This indicates that USP has a significant inhibitory effect on the emission of volatile organic compounds from waste soybean oil recycled asphalt. Among these, the 5% USP sample exhibited the most pronounced inhibition effect, suggesting that at this dosage, the influence of USP on light volatile components reaches an optimal state. When the USP content was further increased to 7%, the total VOC concentration slightly rebounded to approximately 88 mg/m^3^, and the inhibition rate decreased to 32.5%. This decline at 7% USP is attributed to particle agglomeration, which reduces the effective surface area for adsorption, disruption of the continuous physical barrier phase that creates preferential diffusion channels for volatile molecules, and saturation of adsorption sites due to competitive intermolecular interactions among excess USP components.

Moreover, the significant evidence indicates that the VOCs emitted by 8% WSO and samples with different USP dosages were mainly composed of alkanes and hydrocarbon derivatives. Furthermore, the results may demonstrate that alkanes had the highest concentration and appeared to represent the dominant volatile components among the key findings. In light of these results, alkenes ranked second, while the data could suggest that aromatic hydrocarbons consistently showed the lowest content across the study. This indicates that under this temperature condition, the volatile substances released from the waste soybean oil recycled asphalt mainly originate from the light saturated hydrocarbons and some functional group-containing derivatives in the system. As the USP content increased, the concentrations of each component generally showed a decreasing trend, with the most significant decrease observed for alkanes, dropping from approximately 61 mg/m^3^ without USP to about 35 mg/m^3^ at 5% USP; alkenes decreased from about 23 mg/m^3^ to approximately 7 mg/m^3^; hydrocarbon derivatives decreased from about 45 mg/m^3^ to about 35 mg/m^3^; although the overall content of aromatic hydrocarbons was low, they also exhibited a certain degree of reduction.

**Figure 4:**
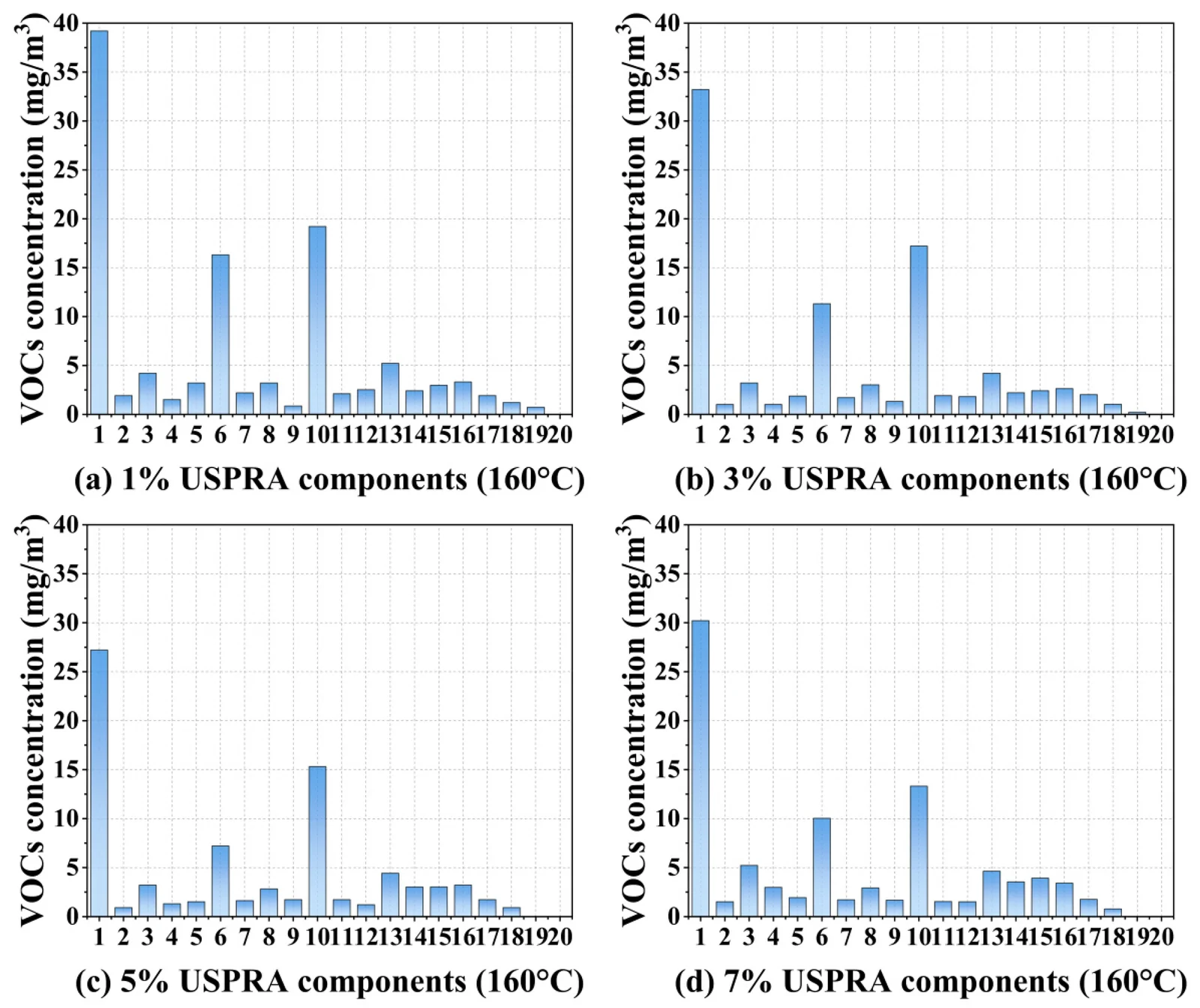
Release patterns of individual VOC components from USPRA at 160 °C.

To further clarify the regulatory effect of USP on the volatile organic compound emissions from 8% WSO recycled asphalt, the main individual VOC components of samples with different USP dosages at 160 °C were analyzed. Table 5 lists the 20 main VOCs emitted from the USPRA asphalt. As shown in Figure 4, the types of VOC components in samples with different dosages were generally consistent, indicating that the addition of USP did not significantly change the composition type of volatiles, but mainly affected the emission intensity of each component. This suggests that the inhibitory effect of USP on VOCs from waste soybean oil recycled asphalt is more reflected in physical retardation and adsorption attenuation of the volatilization process.

From the perspective of the emission intensity of individual components, isopentane, 1-butene, and acetone were always the main released components, with isopentane having the highest concentration, followed by acetone and 1-butene. This indicates that the VOCs released from the system at 160 °C are still mainly low-molecular-weight aliphatic hydrocarbons and some small oxygen-containing organic molecules. This result is consistent with the previous statistical conclusion that “alkanes and hydrocarbon derivatives are dominant,” further demonstrating that light small-molecule components are the main source of high-temperature volatile emissions from waste soybean oil recycled asphalt.

The main peak components demonstrate that increasing USP content from 1% to 7% indicates a clear decreasing trend overall, although the significant reduction magnitudes appear to vary among different compounds. Moreover, the findings might indicate that isopentane concentration gradually decreased from approximately 39 mg/m^3^ at 1% USP to about 30 mg/m^3^ at 7% USP, showing the most notable reduction among the key components examined. This pattern demonstrates that USP exerts a strong inhibitory effect on highly volatile small-molecular aliphatic hydrocarbons. In light of these significant findings, the data may indicate that the relationship between USP content and component reduction appears particularly pronounced for the most volatile compounds in the study. Results show USP inhibits volatile small-molecular aliphatic hydrocarbons strongly. 1-butene decreased from about 16 mg/m^3^ to about 10 mg/m^3^, and acetone decreased from about 19 mg/m^3^ to about 13 mg/m^3^, also exhibiting an obvious downward trend. In contrast, although the other components had lower initial concentrations, they also slightly decreased with increasing USP content, indicating that the inhibitory effect of USP on VOCs is general and not limited to specific types of compounds.

Further comparison of samples with different dosages reveals that the emission levels of most individual components under 5% USP and 7% USP conditions are significantly lower than those under 1% USP and 3% USP, indicating that once the USP content reaches a certain level, its adsorption and migration inhibition effects on volatile small molecules become more effective. However, combined with the previous total concentration analysis, although most individual components under the 7% USP condition remain lower than those in low-dosage samples, the total VOC concentration slightly rebounds compared to that at 5% USP. Thus, although 7% USP still inhibits some components, the overall inhibition efficiency does not continue to improve. This may be attributed to the uneven dispersion of excess USP in the asphalt system, where local agglomeration reduces its effective specific surface area and interfacial interaction capability, thereby weakening its barrier effect on volatile diffusion pathways.

In summary, at 160 °C, the types of VOCs in samples with different USP dosages are essentially the same, indicating that USP does not alter the main composition of volatile products. However, it can significantly reduce the emission intensity of major volatile components, especially low-molecular-weight volatiles represented by isopentane, 1-butene, and acetone. Combined with the observed trends in total concentration and individual components, the inhibition of VOCs from 8% WSO recycled asphalt by USP is primarily manifested as a suppressive effect on light volatile components, with 5% USP exhibiting the best comprehensive regulation effect.

#### 3.1.2. VOC Release Pattern at Different Temperatures

**Figure 5:**
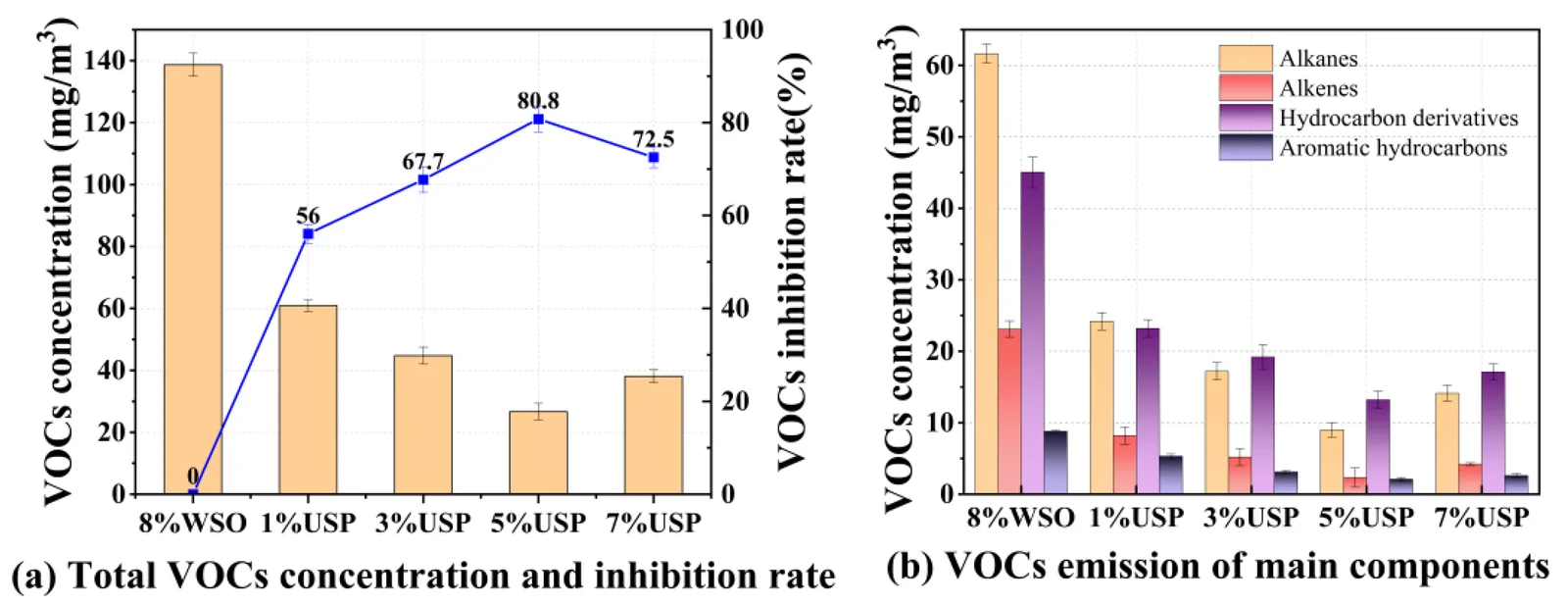
Total VOC emission pattern of USPRA at 140 °C.

At 140 °C, as shown in Figure 5, the total VOC emission concentration exhibited a trend of first significantly decreasing and then slightly rebounding with increasing USP content, consistent with the trend at 160 °C. Without USP, the total VOC concentration of 8% WSO was approximately 139 mg/m^3^. As the USP content increased from 1% to 5%, the total VOC concentration rapidly decreased to approximately 61, 45, and 27 mg/m^3^, with corresponding inhibition rates of 56.0%, 67.7%, and 80.8%, respectively. When the USP content was further increased to 7%, the concentration rebounded to approximately 38 mg/m^3^, and the inhibition rate decreased to 72.5%. Thus, the optimal USP content at 140 °C remains 5%. It should be noted that lower VOC emissions at 140 °C are partly attributable to the intrinsic temperature effect, i.e., reduced vapor pressure and diffusion coefficients of volatile components at lower temperatures. The inhibition rate is calculated relative to the USP-free sample at the same temperature, thereby isolating the USP additive effect from the baseline temperature effect.

Compared with 160 °C, all samples at 140 °C exhibited significantly lower total VOC concentrations and generally higher inhibition rates. At 5% USP, the inhibition rate reached 80.8% at 140 °C, compared to only 39.5% at 160 °C. This temperature-dependent enhancement is attributed to the reduced migration and escape ability of light volatile components at lower temperatures, which allows the adsorption and retardation effects of USP to be more fully utilized. As temperature increases, the accelerated diffusion of light components enables more small molecules to overcome the physical barriers provided by USP, resulting in higher total emissions and lower inhibition efficiency.

In terms of the distribution of various component types, the VOCs at 140 °C were still mainly composed of alkanes, alkenes, hydrocarbon derivatives, and aromatic hydrocarbons, with a composition pattern generally consistent with that at 160 °C, indicating that temperature variation does not alter the compositional characteristics of the main volatile products. However, compared with 160 °C, the concentrations of each component type decreased to varying degrees, with alkanes and hydrocarbon derivatives remaining the main contributors and also the most obvious targets of the inhibitory effect of USP. The higher inhibition rate at 140 °C is primarily because elevated temperatures increase the vapor pressure and molecular mobility of volatile components in the asphalt, generating a larger quantity of VOCs that overwhelm USP’s physical adsorption and barrier capacity, thereby reducing the overall inhibition efficiency. As the USP content increased, the concentration of alkanes decreased the most, demonstrating that low-molecular-weight aliphatic hydrocarbons are always the key components for VOC emission control, whether at 140 °C or 160 °C. Nevertheless, the decrease in each component type was more pronounced at 140 °C, indicating that the restraining effect of USP on light volatile components is stronger at lower temperatures.

**Figure 6:**
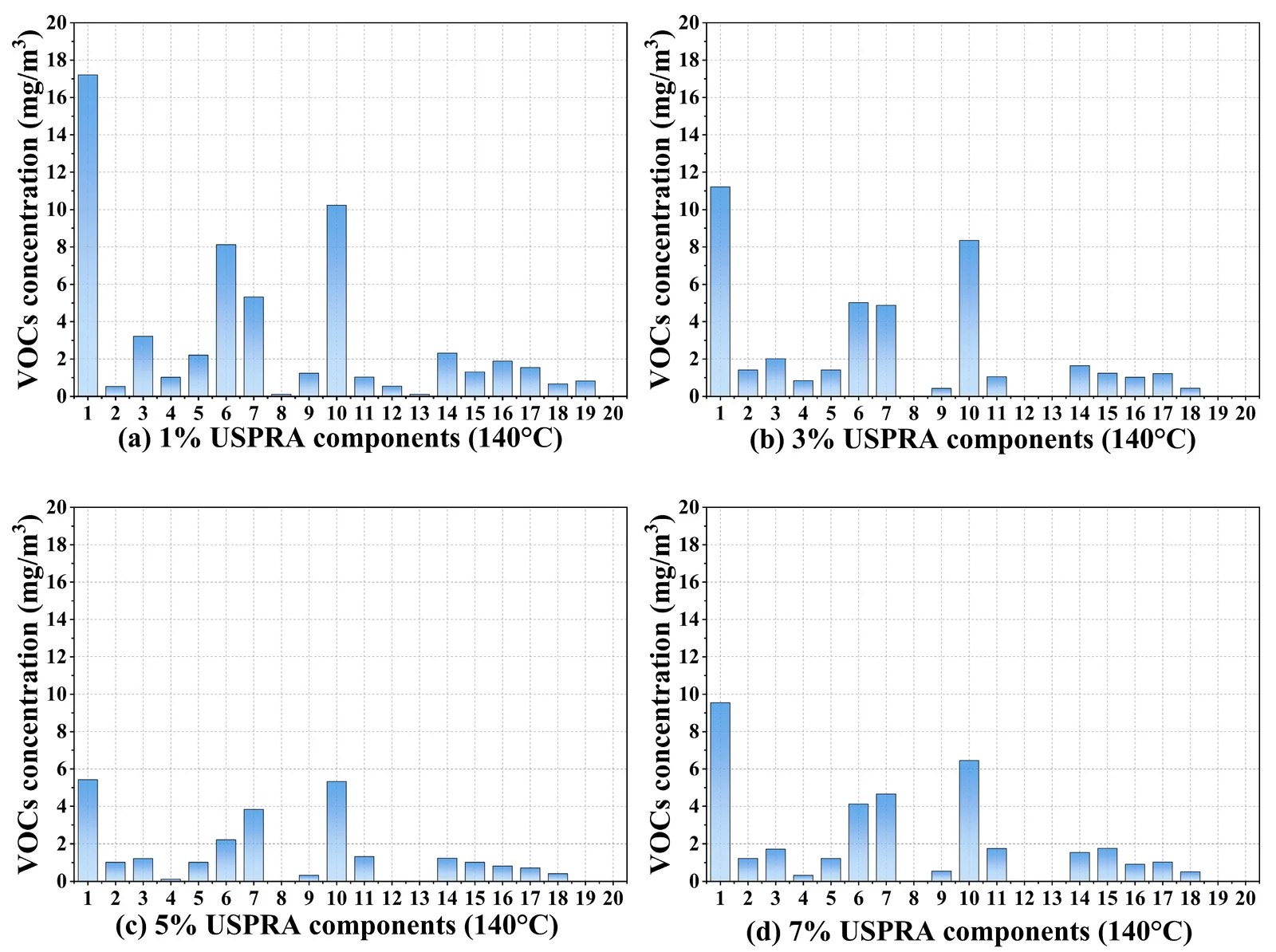
Release patterns of individual VOC components from USPRA at 140 °C.

As shown in Figure 6, at 140 °C, the variation pattern of individual VOC components in samples with different USP dosages was generally consistent with that at 160 °C, i.e., the types of components changed little, and the main difference was reflected in the reduction in emission intensity. Isopentane, acetone, and 1-butene remained the main peak substances in all samples, indicating that the dominant volatiles were still low-molecular-weight aliphatic hydrocarbons and small oxygen-containing organic compounds, which is consistent with the analysis results at 160 °C.

However, compared with 160 °C, the concentrations of the main peak components at 140 °C were significantly lower overall, with particularly notable reductions for isopentane, acetone, and 1-butene, indicating that the escape of light volatile components is more effectively suppressed when the temperature is lowered. Meanwhile, as the USP content increased from 1% to 5%, the main peak intensities continued to decrease, while a slight rebound was observed for some individual components at 7% USP, which is consistent with the previously described “first decrease then increase” trend in total VOC concentration.

Overall, the release patterns of individual components at 140 °C and 160 °C are consistent: both are dominated by light small-molecule components, and the optimal inhibitory dosage in both cases is 5% USP. The difference lies in the fact that at 140 °C, the emissions of the main peak components are lower, and some components do not even appear, indicating that lowering the temperature significantly reduces VOC emissions from USP-modified recycled asphalt.

### 3.2. Investigation of the Mechanism of USP Warm Mix Additive on VOCs

#### 3.2.1. Fourier Transform Infrared (FT-IR) Spectroscopy Analysis

**Figure 7:**
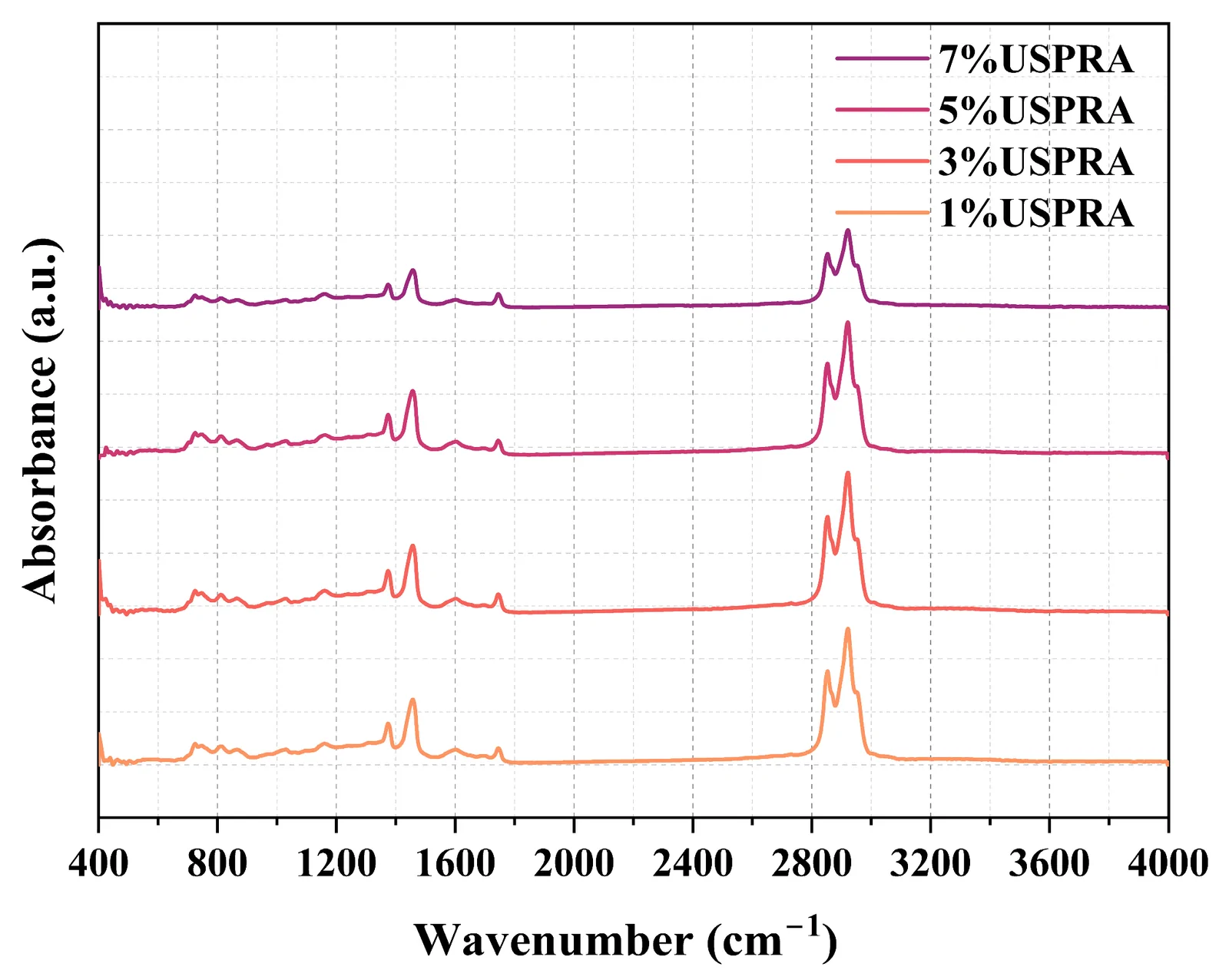
Infrared spectra of USPRA.

Figure 7 demonstrates that the infrared spectra of samples with different USP dosages show consistent patterns. Moreover, the significant spectral peak positions of 1% USPRA, 3% USPRA, 5% USPRA, and 7% USPRA demonstrate that the key functional groups remain generally stable across the results. As USP content increases, the main functional groups remain unchanged, and no new characteristic functional groups appear. Quantitative analysis shows no peak shifts (Δν < 2 cm^−1^) with USP addition, confirming no new chemical bonds. The I_2920_/I_2850_ ratio slightly increased from 1.52 to 1.58, indicating physical conformational adjustments. In light of these findings, the effect of USP on the asphalt system might suggest that physical blending and structural modulation, rather than significant chemical structural reconstruction, could be the dominant mechanism. Functional groups show no new formation.

The absorption peaks near 2920 cm^−1^ and 2850 cm^−1^ correspond to stretching vibrations of aliphatic –CH_2_– and –CH_3_ groups. Therefore, the important absorption peaks near 1450 cm^−1^ and 1375 cm^−1^ may indicate that C–H bending vibrations appear to provide the key evidence. The peak near 1600 cm^−1^ corresponds to the skeletal vibration of aromatic rings. The results in the 700–900 cm^−1^ region show that aromatic ring substitution structures or out-of-plane bending vibrations appear relevant. Additionally, the significant findings from the recycled asphalt samples demonstrate that the key chemical composition framework of the samples under different USP dosages could demonstrate general stability across the data. The samples show stable composition.

Combining the VOC release patterns at 140 °C and 160 °C discussed earlier, it can be observed that although the change in USP content significantly affects the total VOC emission and the peak intensities of major individual components, no new characteristic functional group peaks appear in the infrared spectra, nor is there any significant peak shift. This indicates that the enhancement of the VOC inhibition effect does not originate from fundamental changes in chemical bond types, but is rather attributable to the adsorption, barrier, and binding effects of USP on the migration and escape processes of light components. This finding is consistent with the previous conclusion that “USP does not alter the types of volatiles but mainly reduces their emission intensity.”

#### 3.2.2. Differential Scanning Calorimetry (DSC) Analysis

**Figure 8:**
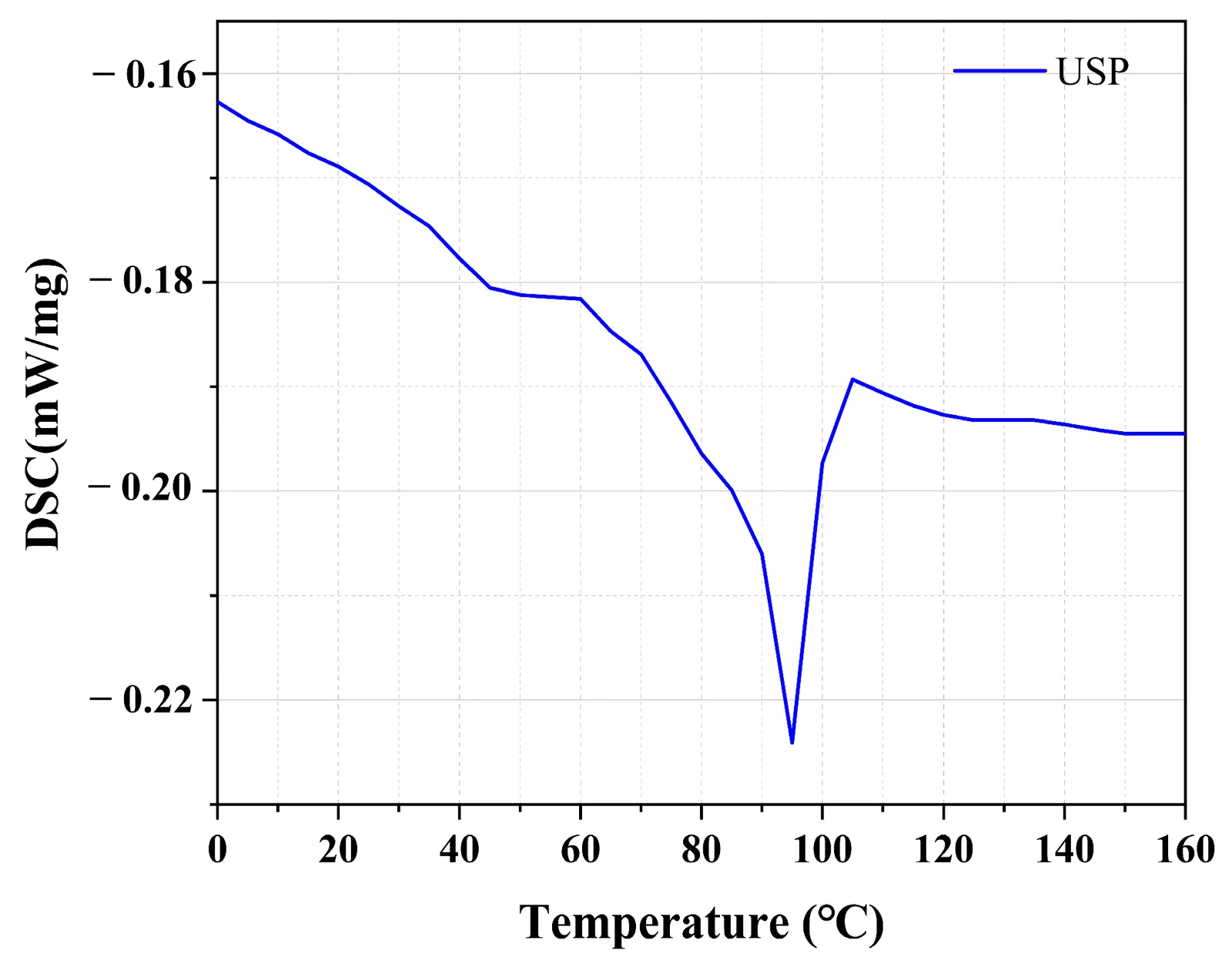
DSC curve of USP.

Figure 8 shows that USP exhibits an obvious endothermic peak during the heating process, with the peak mainly concentrated in the range of approximately 88–99 °C, indicating that USP undergoes a noticeable phase transition within this temperature range, i.e., gradually changing from a solid to a liquid state. The melting enthalpy of USP in the 88–99 °C range is approximately 85 J/g, confirming that USP fully melts at construction temperatures. The VOC inhibition effect is achieved through a cascade mechanism: phase-change melting reduces system viscosity, enabling lower construction temperatures; the reduced temperature lowers vapor pressure and diffusion rates; and molten USP provides physical encapsulation and adsorption, delaying the escape of volatile components.

Further combining the previous FTIR results and the VOC release patterns at 140 °C and 160 °C, it can be seen that the effect of USP does not involve altering the chemical structure of asphalt by generating new functional groups, but rather achieves emission reduction through physical regulation brought about by its phase-change melting characteristics. On one hand, after melting, USP helps to reduce the effective construction temperature of the system; on the other hand, its liquid state facilitates the encapsulation and retardation of light volatile components, thereby inhibiting VOC release. Therefore, from the perspective of thermal properties, it can also be concluded that USP mainly achieves both warm mixing and VOC inhibition effects through a combination of “phase change cooling + physical encapsulation”.

The inhibition of VOCs by USP is primarily governed by physical mechanisms, including phase-change cooling, physical encapsulation, and migration retardation, without altering the chemical structure of the asphalt. On the one hand, USP exhibits a pronounced endothermic phase transition in the range of approximately 88–99 °C, allowing it to fully melt at construction temperatures, thereby reducing the viscosity and effective construction temperature of the asphalt system and minimizing the substantial volatilization of light components. On the other hand, FTIR results indicate that the addition of USP does not introduce new characteristic functional groups, suggesting that it does not alter the system structure through significant chemical reactions. Instead, relying on its molten long-chain structure and good compatibility, USP encapsulates, adsorbs, and retards the diffusion of light alkanes, alkenes, and small oxygen-containing compounds in the asphalt, weakening their migration and escape to the surface. Meanwhile, USP also improves the stability of the asphalt colloidal system to a certain extent, inhibiting the generation of new volatile products during high-temperature oxidation. Consequently, the system exhibits the characteristic of “essentially unchanged component types but significantly reduced emission intensity.” The suppressed VOCs are retained within the system through three mechanisms: physical adsorption onto USP surfaces via van der Waals forces, dissolution into the molten USP organic phase, and diffusion retardation by the USP physical barrier, which prevents escape within the experimental timeframe.

Overall, the inhibition of VOCs by USP is essentially the result of the combined effects of phase-change cooling, physical encapsulation, migration retardation, and structural stabilization, with a more pronounced inhibition effect on low-molecular-weight, highly volatile components. Although phase-change cooling and physical encapsulation are considered the primary mechanisms, the observed inhibition cannot be attributed solely to these factors. Subtle intermolecular interactions, including van der Waals forces and potential hydrogen bonding between the functional groups of molten USP and the polar fractions of volatile compounds, may also contribute to the retardation effect, albeit to a lesser extent.

### 3.3. Effect of USP Warm Mix Additive on the Rheological Properties of Waste Soybean Oil Recycled Asphalt

#### 3.3.1. Temperature Sweep Test Analysis

**Figure 9:**
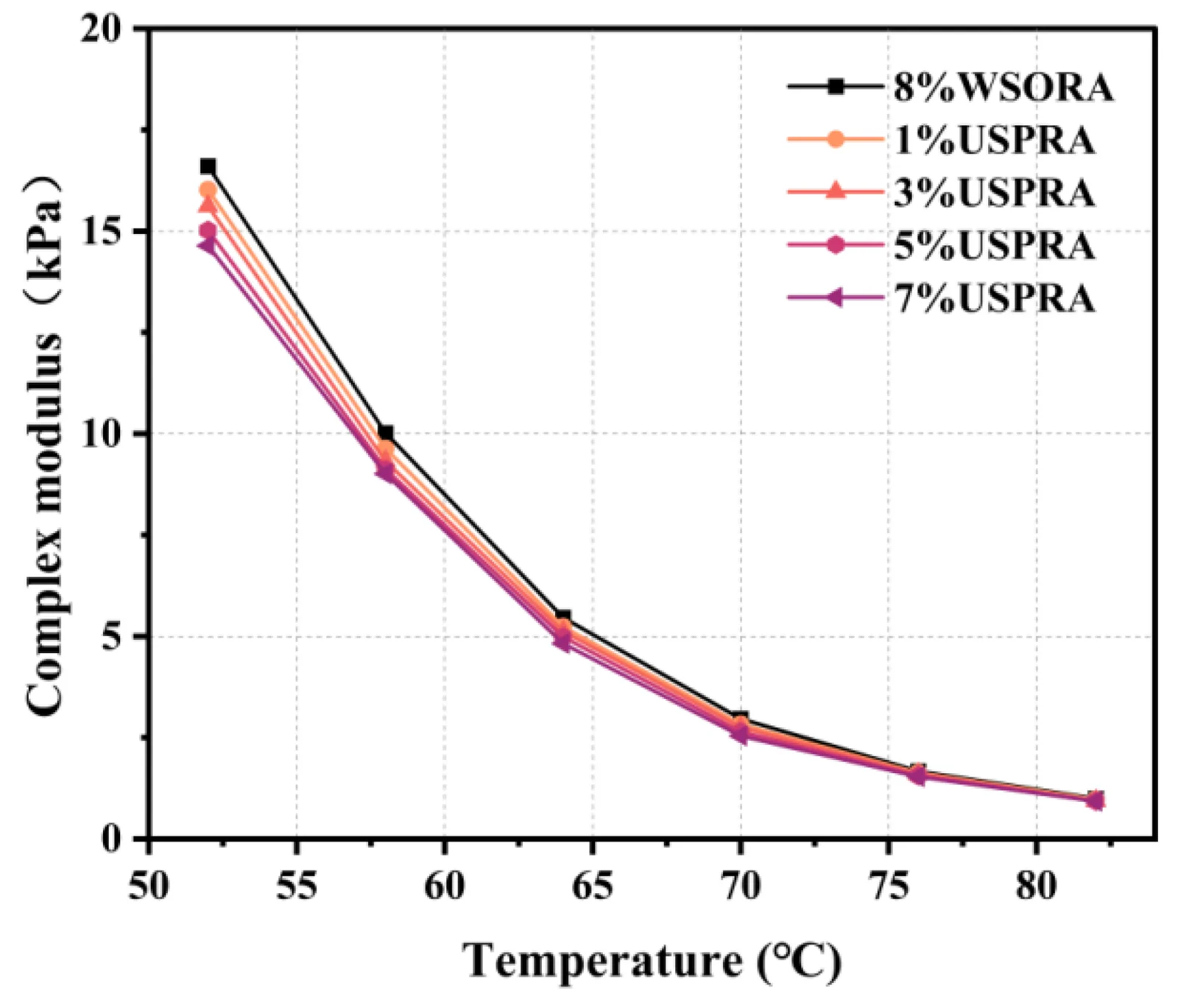
Complex modulus of USPRA.

**Figure 10:**
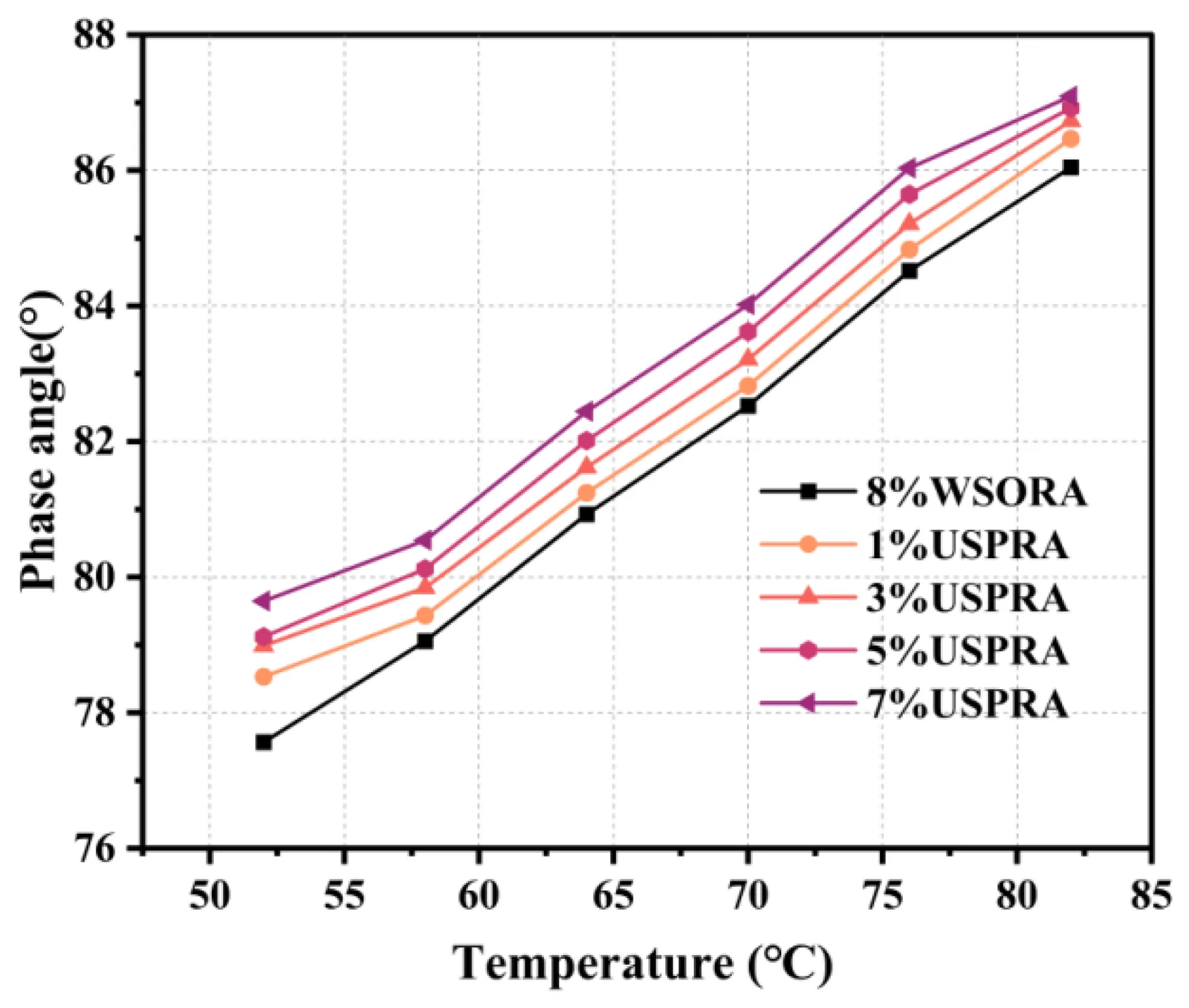
Phase angle of USPRA.

**Figure 11:**
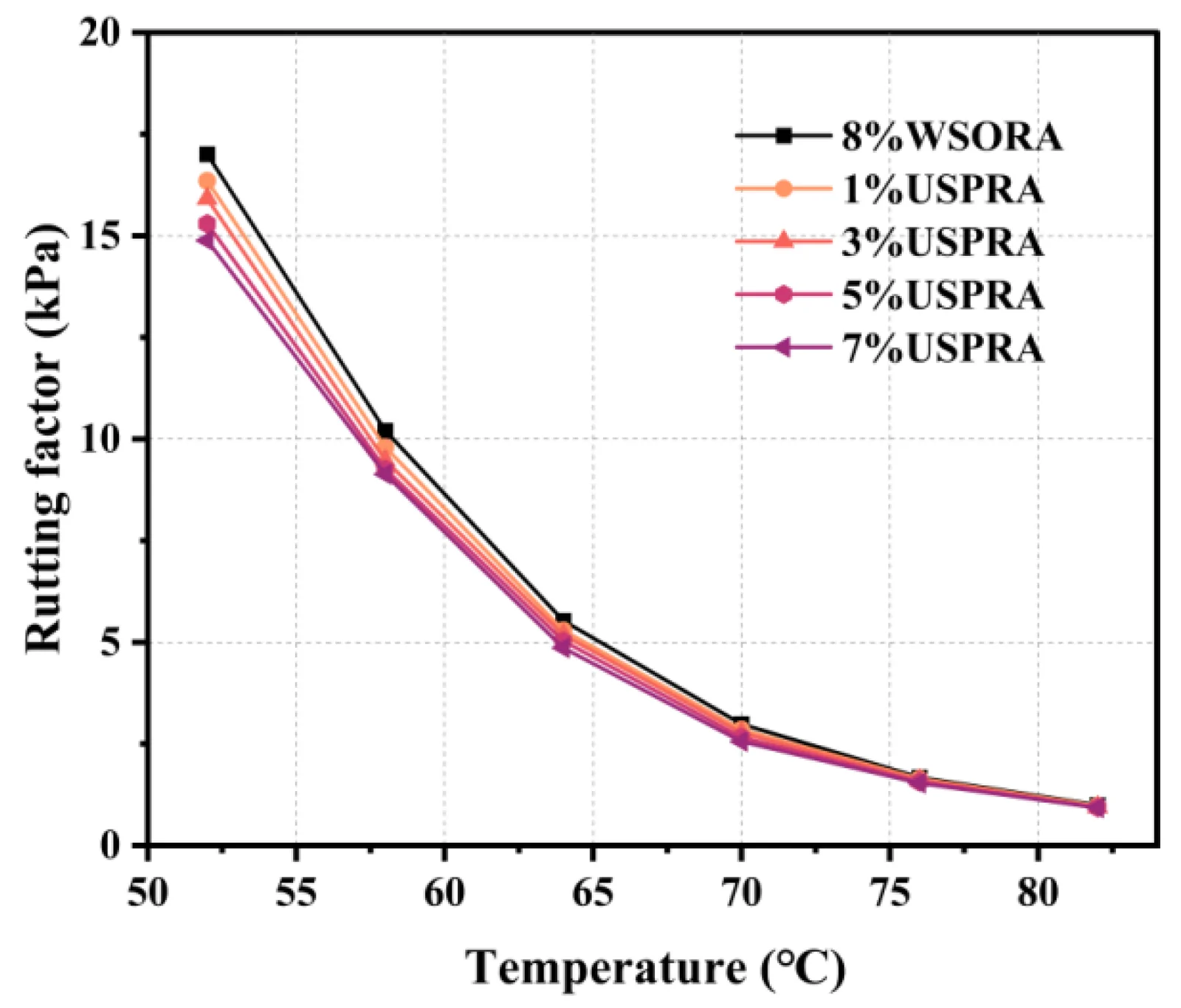
Rutting factor of USPRA.

As presented in Figure 9, Figure 10 and Figure 11, the findings demonstrate that as temperature increases from 52 °C to 82 °C, the complex modulus (G*) and rutting factor (G*/sinδ) of all samples continuously decrease, while the phase angle (δ) gradually increases. Moreover, the significant evidence shows that this temperature-driven increase progressively softens the internal structure of the asphalt system, reduces elasticity, enhances viscosity, and consequently diminishes the high-temperature deformation resistance. Furthermore, the key results confirm that at the same temperature, the G* and G*/sinδ of samples with USP appear generally lower than those of 8% WSO recycled asphalt (8% WSORA), while δ shows a slightly higher value than that of the reference sample. These changes become more pronounced with increasing USP content, indicating that USP reduces stiffness and elastic energy storage, increasing the tendency for viscous flow. Results show that USP softens the asphalt structure, increasing viscous flow tendency. However, the important evidence could suggest that this phenomenon appears consistent with the endothermic phase transition characteristics of USP in the range of 88–99 °C observed in the DSC results. Additionally, the significant findings might indicate that although the temperature sweep range in this test falls below the complete melting range, the oils, organic additives, and resin components in USP could demonstrate a softening and plasticizing effect on the asphalt colloidal system in advance. Thus, the key results demonstrate that this plasticizing effect appears to reduce system viscosity and increase the proportion of viscous components, providing the fundamental reason for the increase in phase angle. Notwithstanding these observations, the critical evidence shows that the interaction between USP and asphalt appears primarily physical, and USP addition may demonstrate that it alters the distribution of saturates, aromatics, resins, and asphaltenes. USP addition is linked to rheological characteristics of looser structure, easier flow, and lower modulus.

The rutting factor (G*/sinδ) may suggest that it serves as a key indicator of a material’s resistance to permanent deformation at high temperatures. Moreover, the significant findings show that the rutting factor of all samples decreases rapidly with increasing temperature, and the evidence appears to show that higher USP content corresponds to a lower overall curve. Furthermore, the results might indicate that USP demonstrates a certain weakening effect on high-temperature rutting resistance. Given that the data could suggest that an appropriate dosage may balance workability and pavement performance, USP’s effect on high-temperature performance appears to remain manageable at moderate levels. Evidence shows USP affects performance. However, the results demonstrate that at low USP dosages, the reduction in the rutting factor appears relatively limited, indicating that a moderate amount of USP could primarily improve workability and reduce mixing temperature. Additionally, the significant results could demonstrate that this moderate dosage has little impact on the original structural skeleton, which may suggest that the key structural integrity remains largely preserved. In light of the evidence, the data might indicate that when the dosage continues to increase, the organic components and softening effect could gradually dominate the system. Notwithstanding these results, the results demonstrate that this dominance weakens the structural stability of the original asphalt system, especially the recycled asphalt, thus demonstrating that the material appears more prone to flow at high temperatures. Research shows increased dosage causes a significant decrease in the rutting factor.

The rheological results may suggest that the two are actually interrelated with the VOC release patterns presented earlier. Moreover, the significant findings show that USP reduces the total VOC emission at 140 °C and 160 °C, with the optimal inhibition range approximately located in the medium dosage range. Furthermore, the rheological figures confirm that the addition of USP makes the system more flowable, increases the phase angle, and decreases the modulus. In light of these results, the evidence may suggest that the effect of USP on the asphalt system is not simply strengthening or weakening, but rather a typical performance balance process. Results show USP reduces construction temperature and inhibits volatilization. However, the significant findings indicate that through phase-change cooling, softening and plasticizing, and physical encapsulation, USP inhibits the volatilization of light components, thereby decreasing VOC emissions. Additionally, the key evidence might suggest that this viscosity-reducing and softening effect also weakens the elastic network and deformation resistance at high temperatures. Given that the results demonstrate that the core of USP application is not to pursue increasingly higher dosages, the findings may indicate that the optimal balance between VOC emission reduction and high-temperature rheological performance is critical. However, excessively high USP content fails to further improve VOC inhibition and may adversely affect high-temperature stability.

The addition of USP may suggest that the complex modulus and rutting factor of the recycled asphalt system decrease while the phase angle increases, indicating that the material exhibits enhanced viscous characteristics. Furthermore, the significant findings show that high-temperature deformation resistance appears weakened as a result of these rheological shifts. In light of the evidence, the results confirm that this change originates from the phase-change viscosity reduction, physical plasticization, and component restructuring effects of USP. Moreover, the key data may suggest that the regulation of recycled asphalt by USP appears to exhibit a dual nature, as the previous VOC analysis indicates that workability improvements and significant emissions reductions occur alongside certain adverse effects on high-temperature rheological properties. USP content affects performance balance. Therefore, the results demonstrate that in practical applications, the USP content should be controlled to establish that a balance between emission reduction and pavement performance appears achievable.

#### 3.3.2. Multiple Stress Creep Recovery (MSCR) Test Analysis

**Figure 12:**
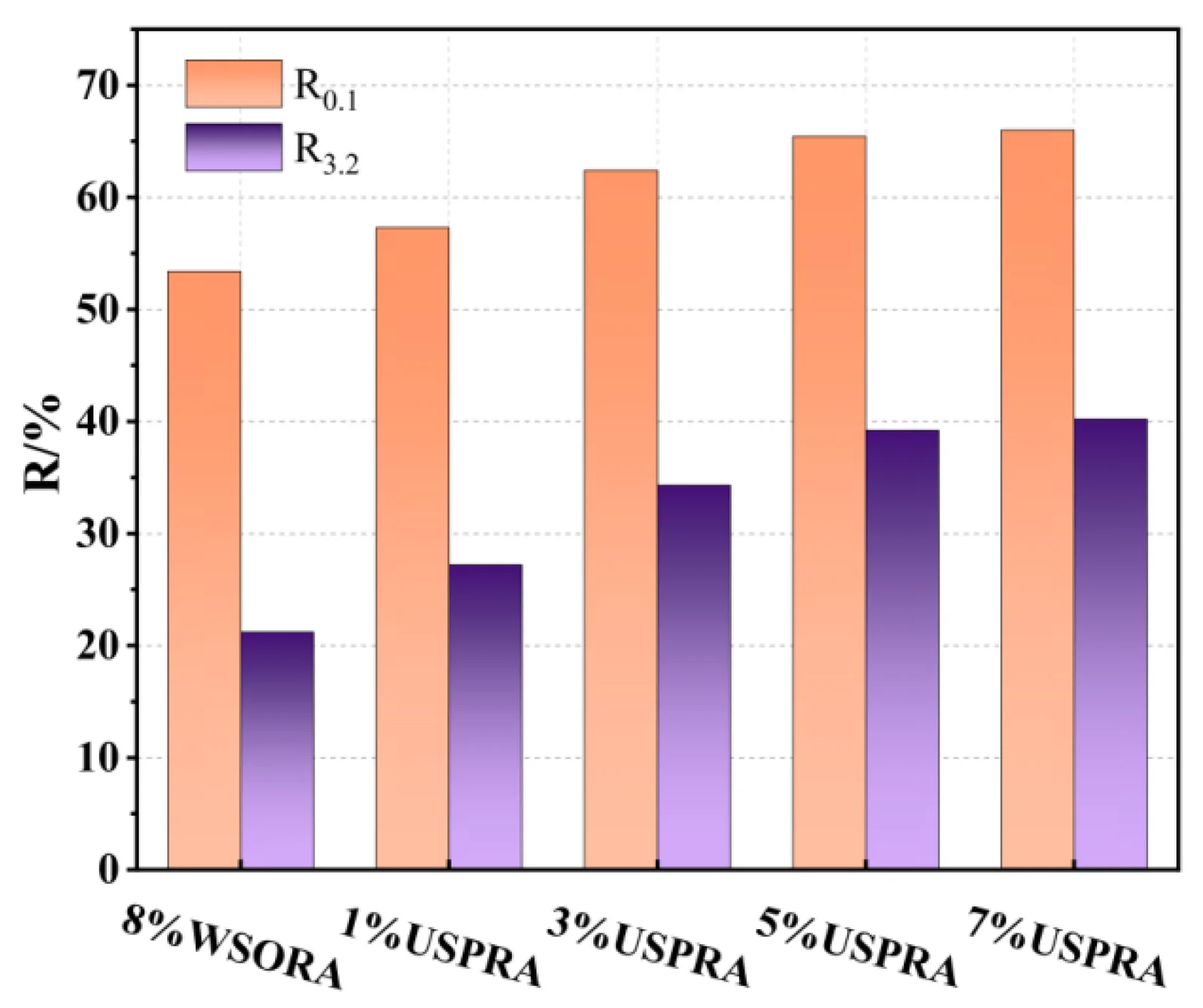
Percent recovery of USPRA.

**Figure 13:**
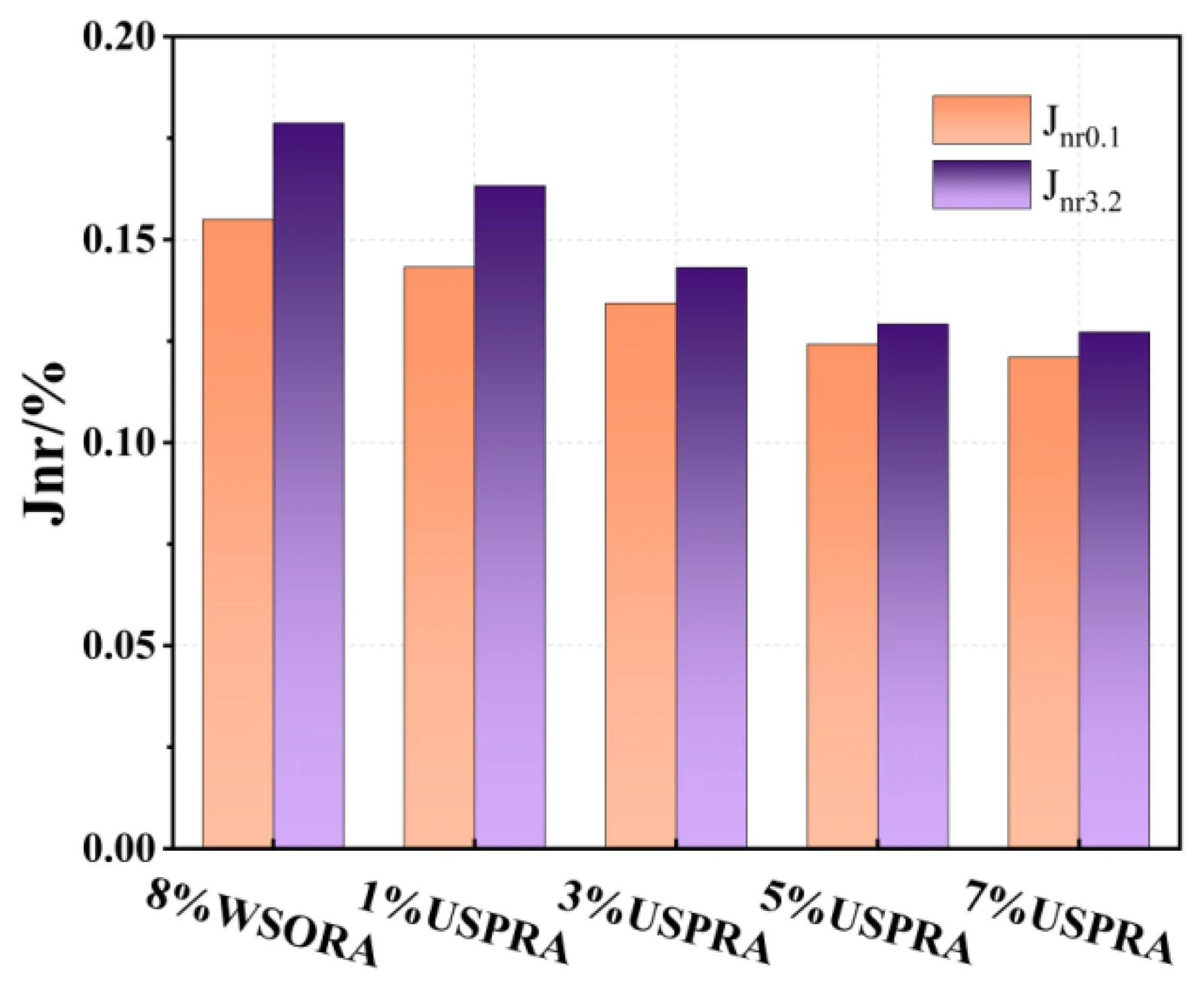
Non-recoverable creep compliance of USPRA.

The results demonstrate that, as shown in Figure 12 and Figure 13, under both stress levels of 0.1 kPa and 3.2 kPa, the percent recovery (R) of all samples shows a general increase with increasing USP content. Moreover, the significant results demonstrate that R_0.1_ increased from approximately 53% for 8% WSORA to about 66% for 7% USPRA, while R_3.2_ increased from about 21% to approximately 40%. Furthermore, the key evidence may suggest that the addition of USP appears to enhance the elastic recovery capacity of the material after unloading, and this improvement could demonstrate more critical effects under high-stress conditions. In light of these findings, the results might indicate that R_3.2_ is consistently lower than R_0.1_, suggesting that under higher stress, the internal structure of the material may appear more susceptible to damage, leading to increased viscous flow and permanent deformation. Non-recoverable creep compliance (Jnr) results show this trend. However, the significant findings could demonstrate that Jnr_0.1_ gradually decreased from 0.155 kPa^−1^ for 8% WSORA to 0.121 kPa^−1^ for 7% USPRA, and the key evidence may suggest that Jnr_3.2_ decreased from 0.178 kPa^−1^ to approximately 0.127 kPa^−1^. Additionally, the important results might indicate that with increasing USP content, the permanent deformation of the recycled asphalt under repeated loading could demonstrate a continuous decrease. Notwithstanding these results, the evidence may suggest that the resistance to permanent deformation appears to be improved with higher USP content. Findings show resistance improves as USP increases. According to AASHTO M 332-22 (S-level traffic), the Jnr_3.2_ requirement is ≤2.0 kPa^−1^. All USP-modified binders satisfy this requirement, with Jnr_3.2_ values ranging from 0.127 to 0.178 kPa^−1^, well below the specification limit. This indicates that despite some reduction in rutting factor, the binders remain suitable for field applications under S-level traffic conditions.

The percent recovery by USP may suggest that dosage variation produces a trend of significant increase followed by leveling off. Moreover, the findings indicate that the increase appears relatively small in the 5–7% range, suggesting that the recovery capacity of the system approaches stability at this point. Given that the previous temperature sweep results demonstrate that USP reduces the complex modulus and rutting factor while increasing the phase angle, the evidence may suggest that USP exhibits a certain viscosity-reducing and softening effect. Furthermore, the significant MSCR results indicate that USP does not simply weaken the high-temperature performance, but rather that it improves the internal elastic recovery process and compensates for part of the adverse effects caused by increased high-temperature fluidity. Results show USP regulation involves synergistic action, not simple softening. However, the key findings may suggest that the molten organic components, resinous substances, and crumb rubber particles promote structural recovery and deformation restoration after unloading. Additionally, the data show that there appears to be an optimal dosage range for USP in recycled asphalt, such that a moderate dosage improves the strain recovery rate. In light of these results, the significant findings might indicate that a moderate dosage also balances the VOC inhibition effect and construction temperature reduction discussed earlier. Notwithstanding the complexity of these interactions, the important evidence may suggest that a USP content of about 5% could demonstrate a reasonable dosage range from the perspective of comprehensive performance. Evidence shows 5% USP balances recovery, VOC inhibition, and temperature reduction effectively. Notably, although 7% USP yields the highest percent recovery and the lowest creep stiffness, its Jnr_3.2_ value (0.127 kPa^−1^) is higher than that of the 5% USP sample (0.112 kPa^−1^), indicating increased susceptibility to permanent deformation under high-stress conditions. Therefore, from the perspective of rutting resistance, the 7% USP dosage is not recommended for field applications, particularly under heavy traffic loads.

#### 3.3.3. Bending Beam Rheometer (BBR) Test Analysis

**Figure 14:**
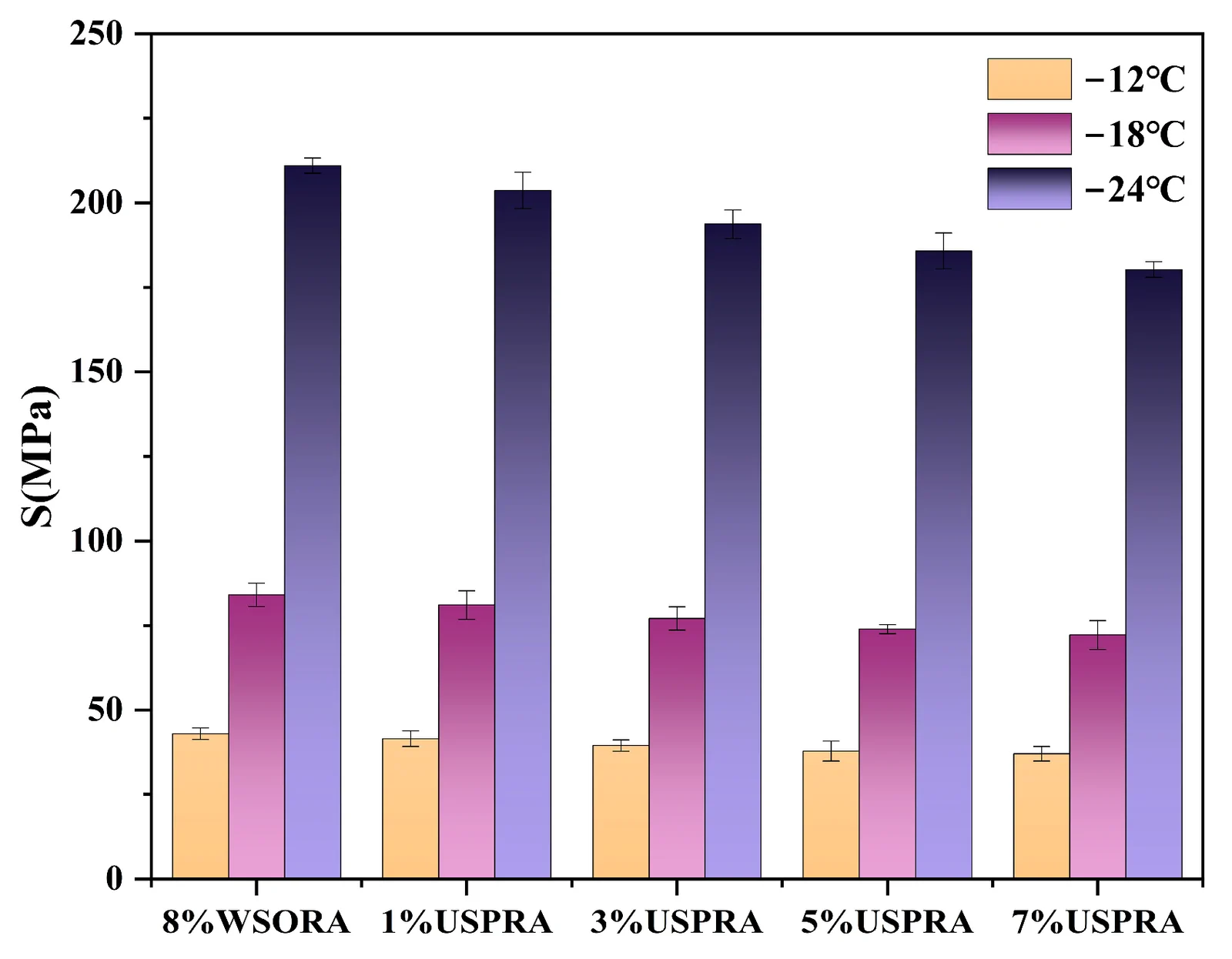
S-value of USPRA.

**Figure 15:**
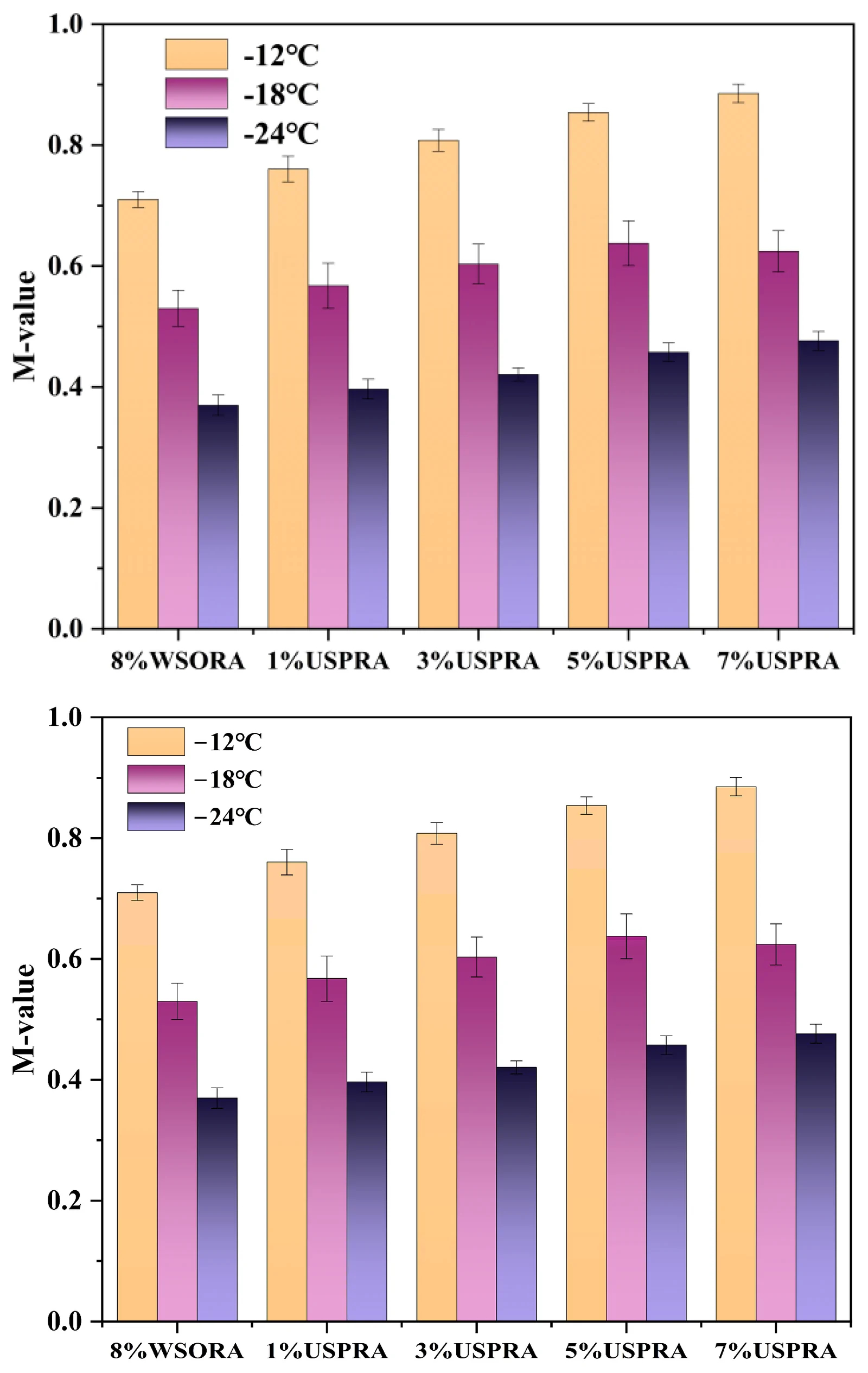
m-value of USPRA.

As shown in Figure 14 and Figure 15, at three temperatures of −12 °C, −18 °C, and −24 °C, the creep stiffness (S) of all samples significantly increases with decreasing temperature, while the m-value generally decreases. This indicates that the lower the temperature, the stiffer the material becomes, the weaker its relaxation capacity, and the higher the risk of low-temperature cracking, which is consistent with the basic low-temperature rheological characteristics of asphalt materials. Under the same temperature conditions, as the USP content increases, the S-value of the samples gradually decreases overall, while the m-value gradually increases. This indicates that the addition of USP can reduce the stiffness of the material at low temperatures, improve its stress relaxation and deformation coordination capacity, and thus enhance low-temperature crack resistance. Among these, the improvement is particularly pronounced at −24 °C, indicating that the regulatory effect of USP on system flexibility is more prominent under more severe low-temperature conditions. From the perspective of dosage variation, the improvement in low-temperature performance by USP generally shows a continuous enhancement, with a more significant increase in the 5–7% range. In particular, 7% USPRA has the lowest S-value and the highest m-value, indicating its best low-temperature crack resistance.

From a mechanistic perspective, this result is consistent with the USP melting characteristics reflected by the DSC results and the trends of decreasing modulus and increasing phase angle in the temperature sweep tests. Thus, organic components and softening agents in USP weaken the brittle structure, promote stress relaxation, and reduce the risk of brittle fracture at low temperatures.

The results may suggest that USP regulation of recycled asphalt could demonstrate obvious dual characteristics. However, the significant findings indicate that USP could effectively inhibit VOC emissions and improve construction performance through viscosity reduction, encapsulation, and retardation. Furthermore, the evidence may suggest that USP could improve low-temperature crack resistance by reducing low-temperature stiffness and increasing relaxation capacity. In light of these results, the data might indicate that USP simultaneously weakens the high-temperature modulus and rutting factor to some extent. The results show USP role in multi-performance balance. Moreover, the key findings could suggest that from the perspective of comprehensive pavement performance, warm mix effect, and emission reduction benefits, USP appears to support a multi-performance balance process. Therefore, the evidence may indicate that this process could demonstrate essential trade-offs, rather than simple enhancement or weakening of a single performance.

## 4. Conclusions

In this study, the VOC emission patterns, inhibition mechanisms, and rheological properties of 8% WSO recycled asphalt modified with different USP dosages (1%, 3%, 5%, and 7%) were systematically investigated. The comprehensive performance analysis indicates that the optimal USP dosage range is 3–5%, with 5% providing the best balance between VOC emission reduction, high-temperature performance, elastic recovery, and low-temperature crack resistance. The main conclusions are as follows:

(1) At 160 °C, the VOC inhibition rate increased from 15.0% (1% USP) to 39.5% (5% USP), then decreased to 32.5% at 7% USP. The optimal inhibition was achieved at 5% USP.

(2) At 140 °C, the inhibition rates for 1%, 3%, 5%, and 7% USP were 56.0%, 67.7%, 80.8%, and 72.5%, respectively. Compared with 160 °C, the inhibition rate at 5% USP increased from 39.5% to 80.8%, demonstrating that lower construction temperatures significantly enhance the inhibitory effect of USP.

(3) FTIR and DSC analyses reveal that VOC inhibition by USP is governed by physical mechanisms, including phase-change cooling, physical encapsulation, and migration retardation. No new functional groups were detected in FTIR spectra, and DSC showed an endothermic phase transition of USP at 88–99 °C with a melting enthalpy of approximately 85 J/g, confirming complete melting at construction temperatures without chemical alteration of the asphalt.

(4) Rheological tests indicate that USP reduces the high-temperature complex modulus (G) and rutting factor (G/sinδ) while slightly increasing the phase angle (δ), indicating some weakening of high-temperature deformation resistance. However, USP simultaneously improves percent recovery (R) and elastic recovery after unloading. Moreover, increasing USP content reduces creep stiffness (S) and increases the m-value, demonstrating enhanced low-temperature crack resistance.

(5) MSCR test results show that R_0.1_ increases from 53% (8% WSORA) to 66% (7% USPRA), and R_3.2_ increases from 21% to 40%. Jnr_0.1_ decreases from 0.155 kPa^−1^ to 0.121 kPa^−1^, and Jnr_3.2_ decreases from 0.178 kPa^−1^ to 0.127 kPa^−1^. These results confirm that USP significantly enhances elastic recovery, with more pronounced improvement under high-stress conditions.

The comprehensive evaluation of VOC emission reduction, rheological properties, and low-temperature performance demonstrates that the regulation of 8% WSO recycled asphalt by USP exhibits distinct multi-performance trade-off characteristics. The results show that USP significantly reduces VOC emissions and improves construction workability through phase-change cooling and physical retardation. At the same time, USP weakens the high-temperature modulus and rutting resistance to a certain extent, while improving elastic recovery capacity and low-temperature crack resistance.

From an engineering economics perspective, the 5% USP dosage achieves an 80.8% VOC inhibition rate at 140 °C, which substantially reduces environmental control costs and health risks during construction. Although USP addition increases material costs by approximately 5–8%, this is offset by energy savings from reduced heating requirements (15–20% fuel reduction), decreased need for fume collection systems, extended pavement service life due to improved crack resistance, and compliance with stringent environmental regulations.

For practical field application, a construction temperature of 140–150 °C is recommended, and a two-stage mixing process (pre-blending USP with recycled asphalt before aggregate mixing) is suggested to ensure uniform dispersion. Based on the rheological results, the USP-modified recycled asphalt is particularly suitable for cold regions, while additional anti-rutting measures may be considered for heavy-traffic hot-climate pavements. The optimal USP content of 5% provides the best balance between emission reduction and pavement performance, offering a cost-effective and environmentally viable solution for sustainable asphalt pavement construction.

## Figures and Tables

**Table 1:** Basic performance indicators of asphalt.

Test	Unit	Standard Range	Test Value
Penetration (25 °C, 100 g, 5 s)	0.1 mm	60–80	71
Softening point	°C	≥46	49
Ductility (15 °C)	cm	≥100	104
Density (15 °C)	g/cm^3^		1.011
Flash point	°C	≥200	315
Solubility (in trichloroethylene)	%	≥99.5	99.85
Viscosity at 60 °C	Pa·s	≥180	201
RTFOT mass change	%	±0.8	−0.06
Retained penetration (25 °C)	%	≥61	66
Retained ductility (10 °C)	cm	≥6	13

**Table 2:** Basic physical properties of waste soybean oil.

Property	Density (15 °C)	Viscosity (25 °C)	Acid Number	Flash Point
Value	0.929 Kg/L	832.5 Pa·s	1.25 mg KOH/g	281 °C

**Table 3:** Performance indicators of USP.

Performance Indicator	USP	Test Method
Appearance	Black	Visual inspection
Physical state	Viscous paste-like fluid	Visual inspection
Density (g/cm^3^)	0.93	JTGE20/T0603
Ash content (%)	≤0.6	Ignition at 525 °C for 30 min
Total organic matter content (%)	≥99.0	Residue after ignition at 525 °C for 30 min

**Table 4:** Conventional performance indicators of aged asphalt.

Test	Unit	Result
Penetration (25 °C, 100 g, 5 s)	0.1 mm	24
Softening point	°C	64
Ductility (15 °C)	cm	184
Density (15 °C)	g/cm^3^	1.15
Viscosity at 60 °C	Pa·s	481

**Table 5:** Main VOC types of USPRA.

No.	Category	VOCs	No.	Category	VOCs
1	Aliphatic hydrocarbon	Isopentane	11	Hydrocarbon derivative	Isopropanol
2	Aliphatic hydrocarbon	1-Pentene	12	Aliphatic hydrocarbon	2,2-Dimethylbutane
3	Aliphatic hydrocarbon	n-Pentane	13	Hydrocarbon derivative	Methacrolein
4	Aliphatic hydrocarbon	n-Hexane	14	Aliphatic hydrocarbon	2-Methylpentane
5	Aliphatic hydrocarbon	Cyclohexane	15	Hydrocarbon derivative	Butanal
6	Aliphatic hydrocarbon	1-butene	16	Aromatic hydrocarbon	Chlorobenzene
7	Hydrocarbon derivative	2,3-Dimethylbutane	17	Aromatic hydrocarbon	Benzene
8	Hydrocarbon derivative	Tetrafluorodichloromethane ^a^	18	Aromatic hydrocarbon	Toluene
9	Hydrocarbon derivative	Chloromethane	19	Aromatic hydrocarbon	Naphthalene
10	Hydrocarbon derivative	Acetone	20	Sulfur-containing compound	Carbon disulfide

^a^ **Compound** No. 8 (tetrafluorodichloromethane) was detected at trace levels (<0.01 mg/m^3^) and may originate from GC-MS background or column artifacts; its presence does not affect the main conclusions.

## Data Availability

The data used to support the findings of this study are included within the article.
